# Removal of *Hsf4 *leads to cataract development in mice through down-regulation of γS-crystallin and *Bfsp *expression

**DOI:** 10.1186/1471-2199-10-10

**Published:** 2009-02-19

**Authors:** Xiaohe Shi, Bin Cui, Zhugang Wang, Lin Weng, Zhongping Xu, Jinjin Ma, Guotong Xu, Xiangyin Kong, Landian Hu

**Affiliations:** 1 Institute of Health Sciences, Shanghai Institutes for Biological Sciences, Chinese Academy of Sciences and Ruijin Hospital, Shanghai Jiaotong University School of Medicine, Shanghai 200025, PR China; 2 State Key Laboratory of Medical Genomics, Ruijin Hospital, Shanghai Jiaotong University, 197 Rui Jin Road II, Shanghai 200025, PR China

## Abstract

**Background:**

Heat-shock transcription factor 4 (HSF4) mutations are associated with autosomal dominant lamellar cataract and Marner cataract. Disruptions of the *Hsf4 *gene cause lens defects in mice, indicating a requirement for HSF4 in fiber cell differentiation during lens development. However, neither the relationship between HSF4 and crystallins nor the detailed mechanism of maintenance of lens transparency by HSF4 is fully understood.

**Results:**

In an attempt to determine how the underlying biomedical and physiological mechanisms resulting from loss of HSF4 contribute to cataract formation, we generated an *Hsf4 *knockout mouse model. We showed that the *Hsf4 *knockout mouse (*Hsf4*^-/-^) partially mimics the human cataract caused by HSF4 mutations. Q-PCR analysis revealed down-regulation of several cataract-relevant genes, including *γS-crystallin (Crygs) *and lens-specific beaded filament proteins 1 and 2 (*Bfsp1 *and *Bfsp2*), in the lens of the *Hsf4*^-/- ^mouse. Transcription activity analysis using the dual-luciferase system suggested that these cataract-relevant genes are the direct downstream targets of HSF4. The effect of HSF4 on *γS-crystallin *is exemplified by the cataractogenesis seen in the *Hsf4*^-/-^,*rncat *intercross. The 2D electrophoretic analysis of whole-lens lysates revealed a different expression pattern in 8-week-old *Hsf4*^-/- ^mice compared with their wild-type counterparts, including the loss of some αA-crystallin modifications and reduced expression of γ-crystallin proteins.

**Conclusion:**

Our results indicate that HSF4 is sufficiently important to lens development and disruption of the *Hsf4 *gene leads to cataracts via at least three pathways: 1) down-regulation of *γ-crystallin*, particularly *γS-crystallin*; 2) decreased lens beaded filament expression; and 3) loss of post-translational modification of αA-crystallin.

## Background

Cataract development is the leading cause of defective vision in humans, and cataracts can be classified as either congenital or age-related. Congenital cataracts account for 10% of cases of childhood blindness, half of which have a genetic cause. More than 20 genes have been implicated in human cataractogenesis [1,2], and these can be divided into two clusters according to the stage of lens development at which they are involved. The first group of genes, including *Pax6*, *Six3*, *Rx*, *Sox2, Pitx3 *and *MAF*, consists of transcription factors at the top of the hierarchy of lens development that are required for the early stages of lens development [3]. Mutations in these genes prevent correct formation of the primary lens fibers, leading to most severe lens defect phenotypes. The second group contains genes that determine lens structure and genes related to structure, including the *crystallins*, *Bfsp*, *MIP*, and the *connexins *[1-3]. Most of the genes identified as causing congenital cataracts fall into the second category. The relationship between transcription factors and lens structural genes warrants further study.

HSF1, HSF2, and HSF4 are members of the heat-shock transcription factor (HSF) family. Only *HSF4 *lacks the carboxyl-terminal hydrophobic repeat (an HR-C domain) that inhibits the formation of active trimers, suggesting a unique functional property [4,5]. Earlier, we found that mutations in *HSF4 *cause autosomal dominant lamellar and Marner cataracts, suggesting a new pathway for cataract formation [6] i, 1997; Tanabe, 1999. Two years later, Smaoui et al. reported that the mutation in intron 12 that causes the exon 12 splicing shift is associated with autosomal recessive total cataracts [7]. Fujimoto et al. and Min et al. reported that disruption of the *Hsf4 *gene caused lens defects in mice, indicating a requirement for HSF4 in fiber cell differentiation during lens development [8,9].

Neither the relationship between HSF4 and crystallins nor the detailed mechanism of the maintenance of lens transparency by HSF4 is fully understood. We used an *Hsf4 *knockout mouse model and a human epithelial cell line to analyze the changes in lens components. Our results indicate that HSF4 regulates a comprehensive array of lens structural proteins. It has a unique role in the development of the lens at the late embryonic and postnatal stages of mouse development; specifically, disruption of the *Hsf4 *gene leads to cataracts via multiple pathways.

## Results

### The Hsf4^-/- ^mouse exhibits aberrant fiber development in the pericentric region of the lens, partially mimicking human *HSF4 *mutation cataracts

**Figure 1:**
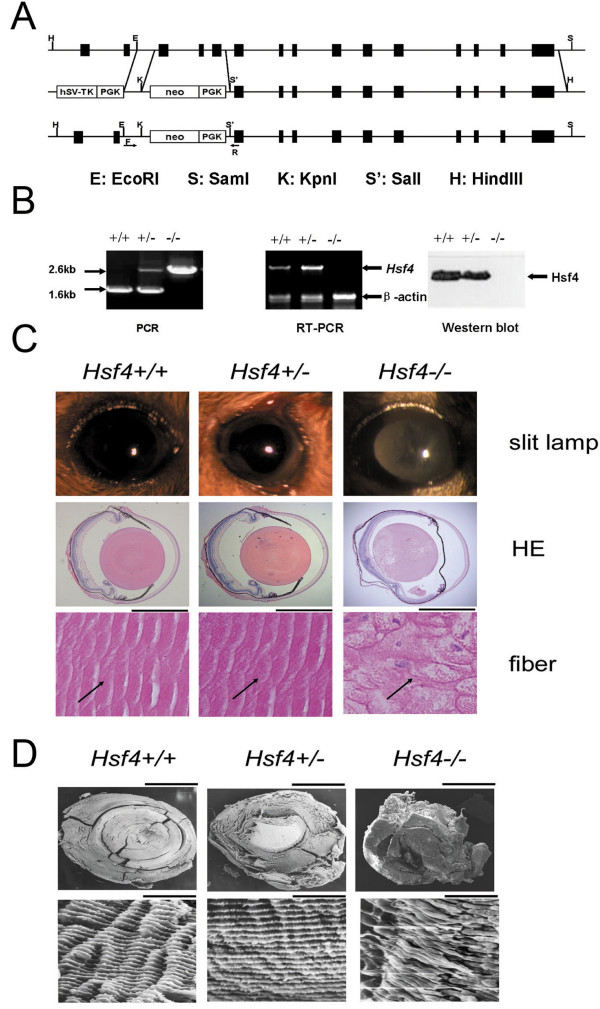
***Hsf4 *knockout results in lens abnormalities and developmental defects, notably swollen and loose fiber structure**. (**A**) The wild-type *Hsf4 *locus, the targeting vector, and the allele following homologous recombination are shown. The targeting vector was used to replace exons 3–5, encoding the DNA-binding domain, with a neomycin resistance gene. (**B**) Analysis of genomic DNA, cDNA, and protein of the *Hsf4 *gene from *Hsf4 *knockout mouse by PCR, RT-RCR, and Western blotting analysis. PCR primers flanking the targeted exons amplified 2.6-kb and 1.6-kb bands in *Hsf4-/- *and *Hsf4+/+ *mice, respectively. Loss of wild-type expression of the *Hsf4 *gene in Hsf4-/- mice was confirmed by the lack of a PCR product after reverse transcription-PCR of total RNA with one of primers within the targeted region. Western blot analysis of lens extracts of 8-week-old mice using a specific antibody against mouse Hsf4 protein showed that Hsf4 protein was absent in Hsf4-/- mice. (**C**) Slit-lamp images of mouse lens and histological examination of lens nuclear region sections of 8-week-old mice. Arrows indicate normal and loose fibers in lens nuclear regions of *Hsf4+/+, Hsf4+/-*, and Hsf4-/- mice. Bar, 50 um. (**D**) SEM images showing the loose and abnormal fibers of *Hsf4 *knockout mice compared with the normal structures of wild-type mice. Bar, 500 um above and 5 um below.

In order to examine the function of HSF4 in lens formation in detail, we generated targeted disruption of the mouse *Hsf4 *gene by homologous recombination of 129S3 embryonic stem (ES) cells. In the vector, exons 3–5, which encode the DNA-binding domain, were replaced with the neomycin-resistance gene followed by the PGK cassette (Figure 1A). The ES cells (129S3 strain, derived from agouti) were electroporated with the linearized targeting vector under positive-negative selection [10], and eight correctly targeting clones were obtained (data not shown). ES cells with correctly targeting clones were injected into a C57Bl/6 blastocyst (black); the genotypes of the offspring were analyzed by PCR to identify wild-type (+/+), heterozygous (+/-), and homozygous (-/-) varieties (Figure 1B). As expected, the ratio of phenotypes was in accordance with Mendelian frequency. *Hsf4*^-/- ^mice are largely normal across all developmental stages except for cataract formation. Under slit-lamp detection, we observed a cataract phenotype in the Hsf4 knockout mouse (Figure 1C). Opacity of the lens of the *Hsf4*^-/- ^mouse appeared at an early postnatal stage and increased with age.

The structure of the *Hsf4*^-/- ^lens fibers became loose, and a vacuole-like cavity that appeared in lens fiber cells at day E15.5 (data not shown) became severe compared to wild-type; undegraded nuclei were clearly seen under a light microscope. However, the bow region of the lens, where the lens epithelial cells differentiate into fibers, was generally normal (Figure 1C). SEM images revealed loose fibers in the lenses of *Hsf4 *knockout mice that showed much less interaction than those of their wild-type counterparts (Figure 1D).

### *HSF4 *has a crucial role in the expression of γ-crystallins, notably γS-crystallin, during postnatal maturation of the lens

**Figure 2:**
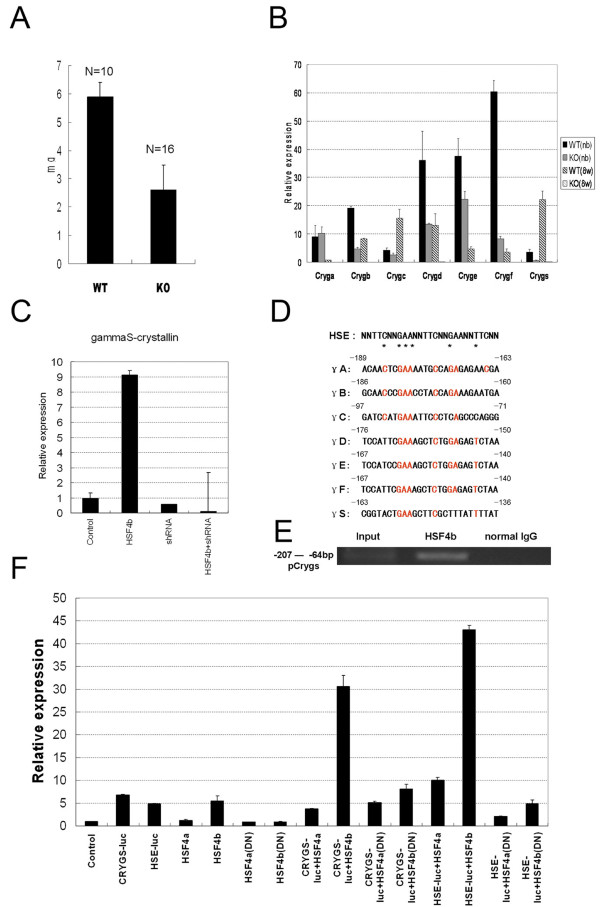
**γ-crystallin expression during lens development**. (**A**) Lens weight in 8-week-old wild-type and *Hsf4 *knockout mice. Ten wild-type lenses and 16 knockout lenses were used to calculate the average weight. (**B**) mRNA levels of all γ-crystallins were analyzed using quantitative RT-PCR. mRNA isolated from lenses in newborns and 8-week-old mice (**C**) Real-time PCR analysis of mRNA levels of γS-crystallin using specific primers. Total RNA was isolated from human lens epithelial cells (SRA01/04) transfected with HSF4b, shRNA for HSF4, or HSF4b plus shRNA for HSF4 respectively. (**D**) Promoter sequence alignment of seven γ-crystallins. Sequences identical to the classic heat-shock element (HSE) sequence are shown in red. Asterisks indicate the key nucleotides essential for heat-shock factor binding. (**E**) CRYGS gene promoter was PCR-amplified from chromatin immunoprecipitation-enriched DNA from human lens epithelial cells (SRA01/04) transfected with HSF4b. (**F**) Relative luciferase activity of the promoter-luciferase construct (CRYGS-luc) by transfection with HSF4a or HSF4b in human lens epithelial cells (SRA01/04). The transfections were performed in hexaplets and the Renilla luciferase plasmid was used as normalization control. The results are shown as means with standard deviations. The classic HSE-luc was used as a positive control.

The *Hsf4*^-/- ^mouse lens is fragile and much lighter, but of a similar size, compared with its wild-type counterpart (Figure 2A). More than 90% of lens proteins are in the soluble form and include a variety of crystallins; in mammals, the crystallins are αA and αB; βB1, βB2, βB3, βA3/A1, and βA4; γA, γB, γC, γD, γE, γF, and γS. In mice, the γ-crystallins are the major contributor to the weight of the mature lens proteins. In humans, β-crystallins and γ-crystallins are in almost equal proportions, with a slightly lower amount of α-crystallins. Fujimoto et al. detected markedly reduced expression of γ(A-F)-crystallin genes in adult HSF4-null mice, even at 2 days old, but the expression levels of γ(A-F)-crystallin genes were normal in the lens of E15.5 HSF4-null embryos. They used chromatin IP (ChIP) analysis to show that HSF4 binds upstream of the γF-crystallin gene, suggesting that HSF4 regulates expression of the γF-crystallin genes [8]. In contrast, Min et al. did not observe a significant reduction of γ-crystallin genes in 1-day-old to 28-day-old Hsf4-null mice, except the γF-crystallin gene, which showed reduced expression in 10-day-old Hsf4-null mice compared to wildtype mice [9]. These conflicting results may be due to differences in Hsf4-null mice construction, differences in the genetic background of the mice being tested, and other unknown factors. However, neither Min et al. nor Fujimoto et al. studied another important crystallin protein, γS-crystallin. During maturation of the mouse lens, the level of γS-crystallin increases fivefold, replacing other types of crystallins and accounting for up to 15% of the total weight [11]. This finding prompted us to examine the expression of γ-crystallins (especially γS-crystallin) in the lens of knockout and wild-type mice. We quantified the mRNA expression of γ-crystallins and assayed the transcriptional activity of *Hsf4*. We found that *Hsf4*^-/- ^mice have reduced expression levels of all subtypes of γ-crystallins directly after birth compared with wild-type mice, and there was almost a complete lack of γ-crystallin expression at 8 weeks old. In wild-type mice, the level of γS-crystallin mRNA indicates that γS-crystallin is only a small proportion of total γ-crystallin at birth, and the level increases to make it the major γ-crystallin at 8 weeks old (Figure 2B). The HSF4 and γS-crystallin genes are first expressed at the late embryonic stages and are up-regulated in late embryonic development [12,13]. The γ(A-F)crystallin genes are located in a gene cluster and are regulated by Sox1 and Maf, but the regulation of γS-crystallin gene (Crygs) has not been well characterized [14-17]. To further evaluate the role of HSF4 in the regulation of γS-crystallin expression, we analyzed the expression of γS-crystallin mRNA in the human lens epithelial cell line SRA01/04 under conditions of *hHSF4b *overexpression [18]. The γS-crystallin mRNA expression level was tenfold higher in *hHSF4b*-overexpressing cells than in the control (Figure 2C). As the γS-crystallin promoter region contains a heat-shock element (Crygs-HSE) that is less conserved than in the promoters of γ(A-F)-crystallin (Figure 2D), we asked whether HSF4b can interact directly with the endogenous promoter of *CRYGS *to regulate its expression. We used ChIP assays to test this hypothesis. The immunoprecipitation of solubilized chromatin prepared from HSF4b-expressing SRA01/04 cells with an anti-HSF4b antibody was followed by PCR using primers that target the potential HSF4b-binding site in the CRYGS promoter region. PCR yielded the expected band in the cells expressing HSF4b, and no band for the normal goat IgG control (Figure 2E). Additionally, we assumed that HSF4b would bind *CRYGS*-HSE to activate γS-crystallin transcription. Under this assumption, the *CRYGS*-luc vector, which contains an upstream sequence of the γS-crystallin gene, was constructed, and luciferase activity levels with and without overexpression of *HSF4b *were determined in the SRA01/04 cell line. The relative luciferase activity with overexpression of *HSF4b *was about sixfold greater than that of the system without overexpression. The dominant-negative *HSF4b*, which had lost the DNA-binding domain of *HSF4b*, had no significant effect on luciferase activity (Figure 2F). These results indicate that *HSF4b *could regulate the expression of γS-crystallin by binding to *Crygs*-HSE.

**Figure 3:**
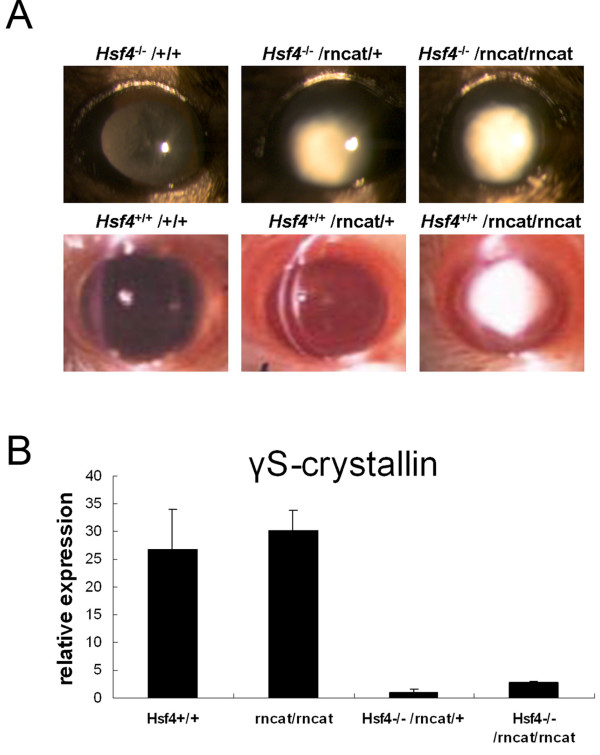
**Intercross of *Hsf4 *knockout mouse and *rncat *mouse**. **(A) **Slit-lamp cataract evaluation of mice with different genotype compositions. Lack of *Hsf4 *induced a more severe phenotype in heterozygous and homozygous *rncat *mice. **(B) **The mRNA levels of γS-crystallin in the *Hsf+/+*, *rncat/rncat*, *Hsf4-/-/rncat/rncat*, and *Hsf4-/-/rncat/+ *mice were analyzed by semi-quantitative RT-PCR.

The γS-crystallin mutant *rncat *is a recessive cataract mouse model with a G to A transition point mutation at position 489 in exon 3 of the γS-crystallin gene [19]. In heterozygous *rncat *mice, the lens is nearly normal. Our results suggested that HSF4 regulates the expression of the γS-crystallin gene (Figure 2). If this is true, lack of HSF4 would decrease the production of the wild-type γS-crystallin and might facilitate the cataract development. As reference, lens opacity of the *rncat *mouse appeared at 11 days old in the nuclear region [19]. Interestingly, when we intercrossed the *Hsf4*^-/- ^mouse with the *rncat *mouse, the offspring (*Hsf4*^-/-^/*rncat/+*) developed cataract in the posterior part of the lens, whereas the lenses of the *Hsf4*^+/+^/*rncat/+ *mice remained basically clear (Figure 3A). The cataract in double null *Hsf4*^-/-^/*rncat/rncat *mice developed earlier, appearing at 7 days old, and seemed more severe than that in *Hsf4*^+/+^/*rncat/rncat *mice (Figure 3A). Lack of Hsf4 worsens the lens fiber defect in γS-crystallin mutation mouse rncat [see Additional file 1]. The mRNA levels of γS-crystallin in the *Hsf4*^-/-^/*rncat/+ *and *Hsf4*^-/-^/*rncat/rncat *mice were reduced by more than 90% compared with the *Hsf4*^+/+ ^and *rncat/rncat *mice (Figure 3B). To certain degree, our results further suggest that *Hsf4 *disruption reduces *Crygs *expression along with the cataract formation in the offsprings in such intercross.

### Intermediate filament genes (*Bfsp1/2*) are down-regulated in the lens of the Hsf4^-/- ^mouse

**Figure 4:**
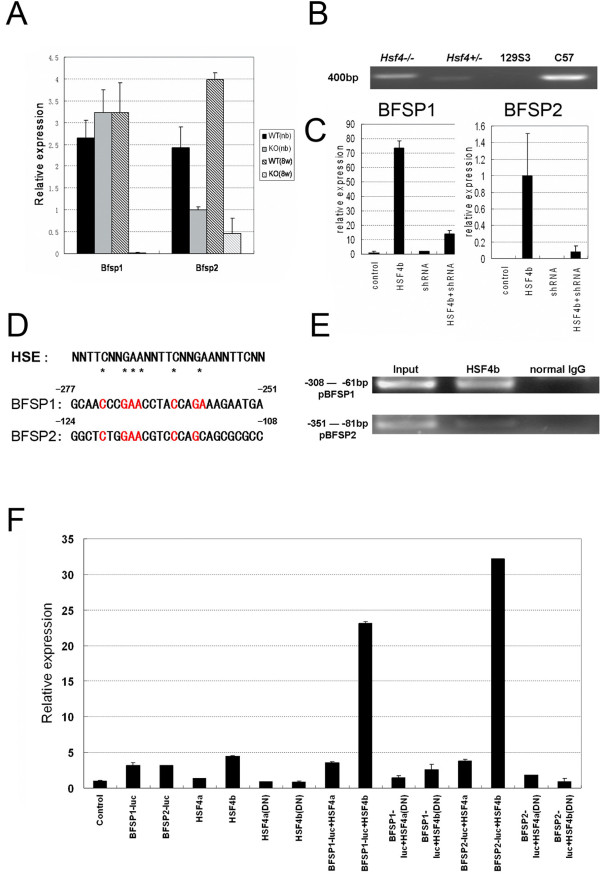
***HSF4 *regulates lens-specific bead filaments *Bfsp1 *and *Bfsp2***. (**A**) Real-time PCR to determine mRNA levels of *Bfsp1 *and *Bfsp2 *in lenses of newborn and adult wild-type and *Hsf4 *knockout mice. **(B) **PCR analysis of *Bfsp2 *gene deletion in *Hsf4*^-/-^, *Hsf4*^+/-^, 129S3 and C57BL/6J mice using primers from the deleted region of the Bfsp2 gene in 129S3 mice. The results suggest that there is at least one copy of the C57BL/6J Bfsp2 allele in the *Hsf4*^-/- ^mouse. **(C) **Real-time PCR analysis of mRNA levels of Bfsp1 and Bfsp2 after transfection of SRA01/04 with HSF4b, shRNA for HSF4, or HSF4b plus shRNA for HSF4. (**D**) Promoter sequence alignment of *Bfsp1 *and *Bfsp2*. Sequences identical to the classic heat-shock element (HSE) sequence are shown in red. Asterisks indicate the key nucleotides essential for heat-shock factor binding. (**E**) Bfsp1 and Bfsp2 gene promoters were PCR-amplified from chromatin immunoprecipitation-enriched DNA from human lens epithelial cells (SRA01/04) transfected with HSF4b. (**F**) Relative luciferase activities of the promoter-luciferase constructs (Bfsp1-luc or Bfsp2-luc) after transfection with HSF4a or HSF4b in human lens epithelial cells (SRA01/04). The transfections were performed in hexaplets and the Renilla luciferase plasmid was used as normalization control. The results are shown as means with standard deviations. The classic HSE-luc was used as a positive control.

An important aspect of *Hsf4*^-/- ^cataract formation is the abnormal development of lens fibers that cannot be explained completely by crystallin regulation. The loose fiber structure of the lens of the *Hsf4 *knockout mouse is similar to that of the *Bfsp1/2 *knockout mouse. Knockout of *Bfsp1 *or *Bfsp2 *resulted in a loose fiber structure with fewer connection proteins among the fibers [20,21]. Therefore, we quantified the levels of *Bfsp1 *and *Bfsp2 *mRNA in the lens of *Hsf4 *knockout mice. At birth, the level of *Bfsp2 *mRNA in *Hsf4 *knockout mice was half that of wild-type mice, whereas the level of *Bfsp1 *mRNA was similar to that of wild-type mice. At 8 weeks old, the levels of both *Bfsp1 *and *Bfsp2 *mRNA in the lens of *Hsf4 *knockout mice were reduced by more than sevenfold compared with wild-type mice, except for the level of *Bfsp1 *mRNA at birth (Figure 4A). It has been suggested that the 129S3 strain mouse has a deletion that causes the loss of exon 2 from *Bfsp2 *mRNA and dramatically reduces mRNA levels of *Bfsp2*. The Bfsp2 protein in this strain was undetectable by antisera to the wild-type protein [22,23]. Since we obtained the expected PCR product size from Hsf4^-/- ^mice using primers from the deletion region of the *Bfsp2 *gene (Figure 4B), there is at least one copy of the C57BL/6J *Bfsp2 *allele in the *Hsf4*^-/- ^mouse. Although *Hsf4*^-/- ^mice carry at least one copy of the highly expressed C57BL/6J Bfsp2 allele, *Hsf4*^-/- ^mice had a much lower level of expression of Bfsp2 compared to the 129S3 wild-type mice. Therefore, lack of Hsf4 results in reduced expression of Bfsp2.

Overexpression of *hHSF4b *in SRA01/04 cells up-regulated transcription of *Bfsp1 *and *Bfsp2*, and this up-regulation could be inhibited specifically by short hairpin RNA (shRNA) targeting *HSF4b *(Figure 4C). As both *Bfsp1 *and *Bfsp2 *promoters have the less-conserved HSE sequence (Figure 4D), we asked whether *HSF4 *can directly bind the promoter region of *Bfsp1 *and *Bfsp2*. We used ChIP assays and detected the expected bands of the promoter sequences of the *Bfsp1 *and *Bfsp2 *genes in cells expressing HSF4b (Figure 4E). This result suggests that HSF4 can bind to the promoter regions of the *Bfsp1 *and *Bfsp2 *genes. We then used the dual-luciferase system to assess whether *HSF4 *activates the expression of *Bfsp1 *and *Bfsp2*. Human *Bfsp1*-luc or *Bfsp2*-luc vectors were co-transfected with or without *HSF4b *into SRA01/04 cells. The relative luciferase activities of *HSF4b *co-transfected with *Bfsp1*-luc or *Bfsp2*-luc were approximately eightfold that of cells transfected with *Bfsp1*-luc or *Bfsp2*-luc alone (Figure 4F). These results indicate that HSF4 may bind specifically to the *Bfsp1*-HSE/*Bfsp2*-HSE sequences and regulate expression of the intermediate filament proteins *Bfsp1 *and *Bfsp2*.

### The 2D electrophoretic analysis of HSF4^-/- ^lens components

**Figure 5:**
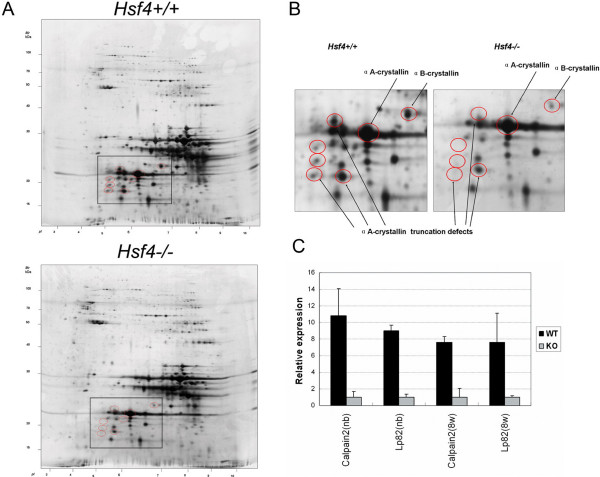
***Hsf4 *knockout mouse lacks lens-specific αA-crystallin modification**. (**A**) 2D electrophoresis map of lens proteins of 8-week-old *Hsf4*^-/- ^and *Hsf4*^+/+ ^mice. There are obvious differences between the two 2D gels in the boxed regions. The most different intensity dots between *Hsf4*^-/- ^and *Hsf4*^+/+ ^mice are indicated with red circles. **(B) **High-resolution images of boxed regions in panel A images showing loss of posttranslational modification αA-crystallin spots in the lenses of *Hsf4*^-/- ^mice. In Hsf4-/- mice, some αA-crystallin truncated fragments were missing in the 2D gel. Proteins were identified by LC/MS sequencing. The arrows point to the truncated αA-crystallins. (**C**) Real-time PCR determining mRNA levels of calpain2 and Lp82 in newborn and adult wild-type and *Hsf4 *knockout mice.

To analyze changes in the lens components of *HSF4*^-/- ^mice, we used 2D electrophoretic (2D-E) analysis to generate maps of lens lysates from 8-week-old *Hsf4*^-/- ^and wild-type (129S3) mice, and identified selected spots using liquid chromatography (LC) with tandem mass spectrometry (MS/MS). By comparison with published 2D-E maps of the mouse lens [11,24], it was possible to identify most of the crystallin proteins in the 2D-E maps of both *Hsf4*^-/- ^and wild-type mice. The lens of *Hsf4*^-/- ^and wild-type mice generally had similar 2D-E protein expression patterns with silver staining. However, alterations of some spots were observed in the 2D-E map of the *Hsf4*^-/- ^lens, which generally had less crystallin protein than the wild-type lens (Figure 5A). We selected and identified some altered spots in the lens map of *Hsf4*^-/- ^mice, where signals were either absent or weaker than in wild-type mice (Figure 5B). Interestingly, 5 of 21 spots were identified by MS as αA-crystallin. These αA-crystallin molecules had a molecular mass of 15–25 kDa and migrated to the acidic region (PI 4.5–6.0) of the 2D-E gel. Other differentially expressed spots were identified as αB-crystallin, βA1-crystallin, βB2-crystallin, γC-crystallin, γB-crystallin, heat shock protein 27, and 7 unknown proteins. Due to the limited amount of sample, the terminal sequences of selected spots were not characterized. A similar pattern has been reported during aging or cataractogenesis in mice [24]. Truncation of αA-crystallin is probably caused by the activation of a class of calcium-activated proteases known as calpains, such as Lp82 or calpain2 [25,26], which have 'clipping' activity *in vitro*. We used real-time PCR to determine the levels of Lp82 and calpain2 mRNAs in the lens of *Hsf4 *knockout mice and of wild-type mice. The mRNA levels of Lp82 and calpain2 in Hsf4 knockout neonatal mice were reduced fourfold compared with wild-type mice; this reduction was even greater in 8-week-old mice (Figure 5C). Therefore, loss of αA-crystallin and reduced expression of Lp82 and calpain2 may be correlated with *Hsf4 *disruption.

## Discussion

The *HSF4 *protein is a member of the heat-shock transcription factor family. Recently, there have been two reports that disruption of the *Hsf4 *gene generated a lens defect, indicating that *Hsf4 *is important for lens cell differentiation [8,9], but the molecular mechanism needs further study. To this end, we generated a *Hsf4*^-/- ^mouse model and analyzed the lens components by molecular biological and biochemical approaches. The roles of γS-crystallin and the lens intermediate filaments Bfsp1 and Bfsp2 in cataract formation in the *Hsf4*^-/- ^mouse were investigated.

Crystallins are a large protein family (including α, β, and γ subtypes) that contains the main lens components. Some crystallins are heat-shock proteins, and most crystallin genes contain an HSE motif, which can specifically be bound by HSF4 in the lens [13]; therefore, down-regulation of crystallins in the lens of the *Hsf4*^-/- ^mouse was expected. However, the levels of the main αA-crystallin components and some β-crystallin components were not significantly altered in the lens of the *Hsf4*^-/- ^mouse at either the mRNA or the protein level [8]. This paradox probably reflects the modulation of crystallins by multiple transcription factors at different stages. *Hsf4*^-/- ^abrogated the expression of γ-crystallins in the lens of mice in the late embryonic stage and postnatally, indicating a time point at which HSF4 functions more independently. In particular, the expression of γS-crystallin seems to be contemporaneous with the postnatal expression of *HSF4 *[12,13], whereas the weight percentages of γ(A-F)-crystallins maintain the same relative distribution throughout development of the mouse lens [11,12]. As expression of γS-crystallin is up-regulated such that it accounts for 15% of the total weight of crystallin protein during lens maturation, the down-regulation of γS-crystallin could be one of the main events caused by *Hsf4 *gene disruption. The role of *Hsf4 *is crucial and cannot be replaced under normal circumstances. Cataract development could therefore be considered a disease state in which there is altered interaction among lens proteins, leading finally to the insolubility of these proteins. In humans, *HSF4 *germline mutations were reported to be associated with recessive adult-onset pulverulent cataract [27] and lamellar cataract that appear late in adult life [6,7]. The data indicate that *HSF4 *plays a critical role in age-related cataract formation. The diversity of phenotypes indicates that the *Hsf4 *mutation family might be involved directly in the development of a background context-dependent cataract.

Another important aspect of *Hsf4*^-/- ^cataract formation is the abnormal development of lens fibers. The development of lens fibers involves multiple cellular processes, including differentiation of lens epithelial cells and fiber cell maturation. The lens of the *Hsf4*^-/- ^mouse exhibited an abnormal, loose fiber structure. It has been reported that HSF4 suppresses the expression of fibroblast growth factor genes which regulate growth and differentiation of lens epithelial cells [8]. A high level of transparency and refraction of the lens is also due to the 'short-range' order between highly concentrated crystallins [28]. Therefore, cataracts can result from changes in protein interactions that are essential for the structure of crystallins. The critical role of protein interactions can be attributed mainly to the lens intermediate filaments Bfsp1, and Bfsp2. These two genes are expressed exclusively in lens fiber cells and can be used as markers of differentiation [29]. The lens fiber structure of the *Hsf4 *knockout mouse is similar to that of *Bfsp1 *or *Bfsp2 *knockout mice [20,21], suggesting that cataract formation probably involves lens intermediate filament deficiency. However, there are also some definite differences, including the looser fiber structure and lack of cellular degeneration in the fiber layers in *Bfsp1/2 *knockout mice. These are apparent in *Bfsp1/2 *knockout mice, strongly suggesting that HSF4 acts through an additional pathway. The short deletion of the *Bfsp2 *gene in the 129 strain [22,23] did not weaken our results; in fact, it enhanced the function of Hsf4. Our Q-PCR analysis revealed that expression of the intermediate filaments Bfsp1 and Bfsp2 is down-regulated in the lens of the *HSF4*^-/- ^mouse. Using dual-luciferase analysis, we further identified these two genes as possible direct targets for regulation by *HSF4*. Down-regulation of lens intermediate filament proteins caused by lack of *HSF4 *could be an important aspect of abnormal differentiation and continued fiber cell maturation. High levels of transparency and refraction of the lens are due to the short-range order among highly concentrated crystallins [28]. The critical role of this protein interaction is attributed mainly to the lens intermediate filament proteins *Bfsp1 *and *Bfsp2*. The *Bfsp2 *mutation is associated with cataract, suggesting it has an important role in formation of the lens fiber [30,31]. These proteins have been proposed to function in the assembly and connection of crystallins [32]. These proteins form a thin filament backbone that is decorated at regular intervals among mature fibers. Bfsp1 and Bfsp2 are the main components of this backbone [33,34].

Over a lifetime, lens crystallins undergo a wide variety of irreversible and covalent modifications by processes such as proteolysis, deamidation, and oxidation. These post-translational modifications are often considered the cause of changes in protein interactions, resulting in reduced solubility of crystallins in the aging lens [35]. We characterized the expression of *Hsf4*^-/- ^lens proteins using 2D-E and subsequent MS. These modifications were attributed to activation of a class of calcium-activated proteases called calpains, including Lp82 and calpain2. Lp82 co-localizes with crystallins in the lens fiber cell cytoplasm and may be important for the truncation that occurs during lens maturation and for maintenance of crystallin architecture. Calpain2 is more likely to be involved in cell-signalling pathways, as suggested by its localization in the epithelium and peripheral fibers of the lens [36]. We observed that expression of Lp82 and calpain2 is reduced during lens development in the *Hsf4 *knockout mouse. This potentially indicates a novel role for *HSF4 *in lens development.

## Conclusion

We have determined a high hierarchical position for *HSF4 *in the regulation of lens formation, and we have assessed its unique, important regulatory effects during the later stages of lens maturation. This regulation is active specifically in the formation of delicate fiber structures that eventually contribute to lens development and the maintenance of transparency. Maintaining the delicate microscopic structure and transparency in a systematic manner, the lens can be considered to be constructed of crystallins connected by intermediate filament proteins and shaped by lens proteolysis systems in the *HSF4 *regulatory system.

## Methods

### Generation of the *Hsf4 *knockout mouse

A clone containing the entire mouse *Hsf4 *gene was isolated from a 129S3 mouse genomic library (Genome Systems). From this, a 2.8 kb 5'-flanking region *Eco*RI/*Kpn*I fragment containing part of the promoter region was subcloned into X-pPNT. In X-pPNT, exons 3–5 and part of the promoter region were replaced by a 1.6 kb neomycin-resistance gene fragment. A downstream 5.0 kb *Sal*I/*Not*I fragment was inserted into X-pPNT. These linearized segments were inserted into X-pPNT to generate pPNT-HSF4, which was linearized with *Eco*RI/*Not*I and electroporated into R1 ES cells. Two ES clones with a targeted disruption were identified from 131 clones isolated by the positive-negative G418/gancyclovir selection method. Targeted clones were identified by PCR and Southern blot analysis after *Hind*III digestion of genomic DNA, using a flanking probe 3' to the replacement. The ES clones were microinjected into C57Bl/6 blastocysts, and five chimeric mice were produced. These chimeras were bred with C57Bl/6 mice to generate progeny heterozygous for the mutation. Heterozygous mice were crossed to produce *Hsf4*^+/+^, *Hsf4*^+/-^, and *Hsf4*^-/- ^mice. Germline transmission of the mutations was determined by PCR analysis of tail DNA, as described above. All animal experiments were conducted under protocols approved by institutional Animal Care and Use Committees and according to the National Institutes of Health "Using Animals in Intramural Research" guidelines.

### Histopathology

Mouse eyes were dissected, fixed in 4% paraformaldehyde for at least 2 weeks, embedded in paraffin, and cut into 6 μm thick sections. These paraffin-embedded sections were stained with hematoxylin and eosin.

### Scanning electron microscopy

The mice were sacrificed, the eyes were enucleated, and the corneas were removed. The remaining orbit was immediately placed into a fixative of 2% glutaraldehyde in 0.1 M sodium cacodylate buffer at pH 7.4. Tissues were fixed at 4°C for 2–3 days, with daily changes of the fixative. After washing overnight in 0.2 M sodium cacodylate buffer, the lens was removed from the posterior portion of the eye containing the retina. To expose the fibers and suture patterns, the lens capsule and superficial fibers were peeled away from the lens around its diameter. Both the fiber peelings and the remaining lens core were collected and processed for scanning electron microscopy (SEM). Specimens were post-fixed in 1% aqueous OsO_4 _at 4°C for 12 hours and washed in 0.1 M sodium cacodylate buffer at pH 7.4. They were dehydrated in a series of graded ethanol and processed by critical point drying. A layer of gold 20 nm thick was deposited onto the surface of the dried samples, which were then examined using a Hitachi S-520 scanning electron microscope at 20 kV.

### Reverse transcription and quantitative real-time PCR analysis

RNA was isolated using a Qiagen RNA-easy Mini Kit (Qiagen). A 1 μg sample of total RNA was reverse transcribed at 42°C for 1 hour in a total volume of 20 μL (Promega), with oligo(dT)_15 _primers. At the end of the reaction, the mixture was heated at 70°C for 15 minutes to inactivate the reverse transcriptase. For Q-PCR (Table 1), 1 μL of cDNA was amplified for 45 cycles with a master mix (SYBR Green Supermix; ABI) using a thermocycler (MG). Melting curve analysis was done at the end of the reaction (after 45 cycles) to assess the quality of the final PCR products. The threshold cycle C(t) values were calculated by fixing the basal fluorescence at 0.05 unit. Five replicates were used for each sample, and the average C(t) value was calculated. The ΔC(t) values were calculated as C(t) sample – C(t) β-actin. The N-fold increase or decrease in expression was calculated by the ΔΔCt method using the fetal C(t) value as the reference point. The N-fold difference was determined as:

**Table 1:** PCR primer pairs.

Gene name	Primers	Annealing temperature
*hHSF4F*	CCCCCTCAAGAAAGTGTGGAA	60°C
*hHSF4R*	GAACCAAGGGCTGCATCAAG	60°C
*mHSF4F*	GGCACCAGCTTCCTCGTAAG	60°C
*mHSF4R*	CCTTCCGAAAACCATACATGTTG	60°C
*hCrygsF*	CCCAACTTTGCTGGGTACATG	60°C
*hCrygsR*	GGCCTCCACTAGGCAGATGA	60°C
*mCrygsF*	AGCGTTGGATGGGCCTTAAT	60°C
*mCrygsR*	GTTTCGTACATCTGACCGTTGAAG	60°C
*hBFSP1F*	TTCAGTGGGAGAGAGATGTTGAAA	60°C
*hBFSP1R*	CTCCTGGGTCTGGGACATGT	60°C
*mBfsp1F*	AGACGCCCATCTCTCTGATCA	60°C
*mBfsp1R*	AGGGCCTTCTGTCTCGGTTT	60°C
*hBFSP2F*	TTCAGTGGGAGAGAGATGTTGAAA	60°C
*hBFSP2R*	CTCCTGGGTCTGGGACATGT	60°C
*mBfsp2F*	GCACCGGTCTGGATGATGTC	60°C
*mBfsp2R*	ACGTGGACCACCTCTGTCTG T	60°C
*mLp82F*	ATTGGCTTCGCCATCTACGA	60°C
*mLp82R*	CCCGCATGTTGATGTAGGTTT	60°C
*mCalpain2F*	ATGCGGAAAGCACTGGAAGA	60°C
*mCalpain2R*	CCAAACACCGCACAAAATTG	60°C

2^-(ΔC(t)sample-ΔC(C(t)fetal)^

### SDS-PAGE and Western blot analysis

Electrophoresis of extracts was done under denaturing reducing conditions using 4%–12% Nupage Bis-Tris gels with Mops-SDS running buffer (Invitrogen). Approximately 30–50 μg of protein was used per lane for analysis as described in the protocol. Electrophoresis was for approximately 35–60 minutes at 200 V. After electrophoresis, the proteins were transferred to nitrocellulose membranes (Amersham) using the Surelock X-cell transfer system (Invitrogen). The blotted membranes were labelled with antibodies against *HSF4 *or *HSF4b *(Santa Cruz). The blots were washed in TBS/T, incubated using the ECL kit (Cell Signaling Technology. Inc.), and exposed to X-ray film (Kodak).

### Cell culture, transfection, and luciferase assay

Human lens epithelial cells (SRA01/04) were grown and cultured in Dulbecco's modified Eagle's medium (DMEM) with low glucose (1 mg/ml) and supplemented with 15% fetal bovine serum, gentamicin (50 units/mL; Fluka), and a penicillin-streptomycin antibiotic mix (50 U/ml; PAA) at 36.5°C in an atmosphere of 5% CO_2_. The methods for establishing primary cultures of human lens epithelial cells were as described [18]. Cells from explant cultures were dissociated using trypsin-EDTA solution (PAA) and collected by centrifugation. The cells were subcultured in the initial medium at 36.5°C in a humidified atmosphere with 5% CO_2_. The procedure was repeated for additional subcultures.

Cells were cultured as described above. Transient transfections were done using FuGENE 6 reagent (Roche) essentially according to the manufacturer's protocol but with minor modifications. SRA01/04 cells were plated in six-well dishes of regular medium at a density of 2 × 10^6 ^cells/ml. A 2 μg sample of plasmid DNA was prepared in 100 μl of serum-free, antibiotic-free medium and incubated with 6 μl of FuGENE 6 reagent prepared separately in 100 μl of serum-free, 15% FBS medium. After 30 minutes, the DNA mix was added to the cells, which were then incubated for 24 hours at 37°C. The cells were washed in phosphate-buffered saline, supplemented with DMEM with 15% FBS, and incubated overnight at 37°C. In co-transfection experiments, the appropriate empty vector DNA was used to ensure similar concentrations of DNA under all conditions. In all transfections, 0.1 μg of *Renilla *luciferase plasmid (Promega, Madison, WI) was included to control for transfection efficiency. Protein concentration was determined by the Bradford assay. Firefly and *Renilla *luciferase activities were quantified using the Dual Luciferase kit (Promega), according to the manufacturer's instructions. The *Renilla *luciferase values were used for normalization of differences in transfection efficiency. The normalized values were used to calculate the ratio of firefly luciferase activities in induced and non-induced cells (induction factor).

### Chromatin immunoprecipitation (ChIP) assays

Preparation of chromatin DNA from SRA01/04 cells expressing HSF4b or control plasmid pCDNA3.1(+) and subsequent ChIP assays were done with a chromatin immunoprecipitation assay kit (Upstate Biotechnology, Waltham, MA) following the manufacturer's instructions. HSF4b/DNA complexes were precipitated by anti-HSF4b antibody. Precipitated DNA was amplified by PCR using primers flanking the potential HSF4-binding site (positions -207 to -64) in the CRYGS promoter, positions -308 to -61 in the Bfsp1 promoter, or positions -351 to -81 in the Bfsp2 promoter. Goat IgG was used as the negative control. The primers are given in Table 1. The PCR products were resolved by electrophoresis in a 1.5% agarose gel.

### 2D electrophoresis and identification of lens proteins

Ten lenses from identical 8-week-old mice were homogenized by sonication in 1 ml of lysis solution containing a protease inhibitor cocktail (9.8 M urea, 2% Chaps, 1% DTT). The protein concentration was measured by Bradford assay, using bovine serum albumin as the standard, and samples were stored at -70°C.

Isoelectric focusing was done using immobilized pH gradient (IPG) gel strips (13 cm, pH 3–10), followed by molecular mass-based resolution by SDS-12% PAGE (Amersham). The silver-stained gel images were captured and image analysis was used (Imagemaster, Amersham) to determine the contribution of each spot to the total protein. The isoelectric point (pI) of each unmodified crystallin was extrapolated using the calculated pI and position as references. The spots captured from a Coomassie brilliant blue-stained gel were washed, dried, and digested with trypsin; then LC-MS/MS was used to determine the amino acid sequence of the peptides.

## Abbreviations

HSF4: heat shock transcription factor 4; SEM: Scanning electron microscopy; ChIP: Chromatin immunoprecipitation

## Authors' contributions

XS carried out most molecular biology experiments; BC and ZW generated the HSF4 knockout mouse; XS, ZX and JM characterized the phenotypes of HSF4 knockout mouse; XS, GX and LW prepared the manuscript; XK and LH conceived of the study, and were responsible for its design and coordination; LD edited the final version of the manuscript. All authors read and approved the final manuscript.

## Supplementary Material

Additional file 1 **Lack of *Hsf4 *worsens the lens fiber defect in γS-crystallin mutation mouse rncat.** Hematoxylin and eosin stain revealed an aggravated lens fiber defect in 8-week-old heterozygous and homozygous rncat mice in the absence of the *Hsf4 *gene. Bar, 50 um. Click here for file
