# Supplementary material for: Removal of Hsf4 leads to cataract development in mice through down-regulation of γS-crystallin and Bfsp expression
Source: BMC Mol Biol. 2009 Feb 19;10:10. doi: 10.1186/1471-2199-10-10 (PMC2653017; doi:10.1186/1471-2199-10-10)
Supplement: Additional file 1 — Lack of Hsf4 worsens the lens fiber defect in γS-crystallin mutation mouse rncat. Hematoxylin and eosin stain revealed an aggravated lens fiber defect in 8-week-old heterozygous and homozygous rncat mice in the absence of the Hsf4 gene. Bar, 50 um. [file 1471-2199-10-10-S1.doc]

Additional file 1. Lack of *Hsf4* worsens the lens fiber defect in γS-crystallin mutation mouse rncat.


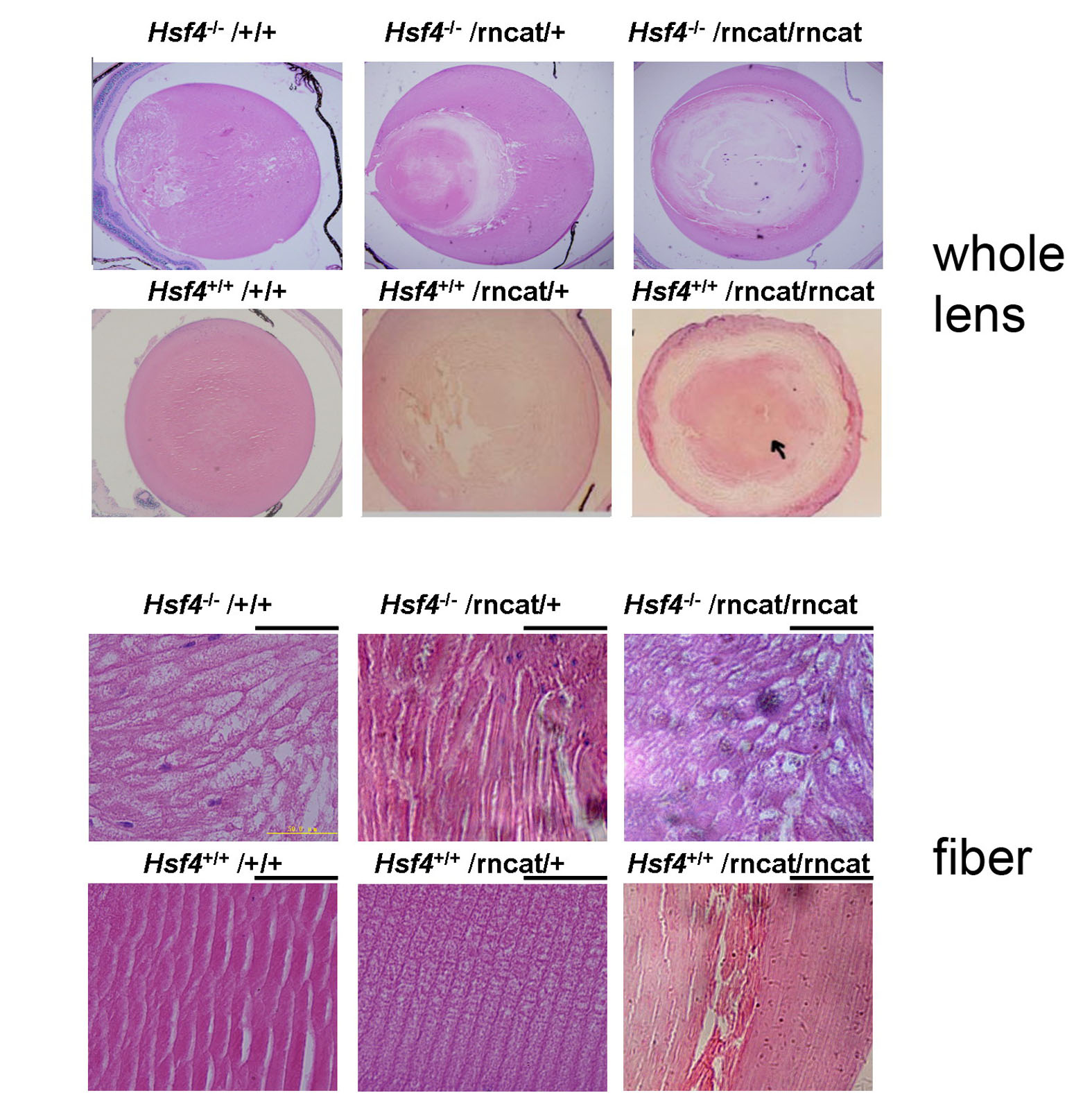


Hematoxylin and eosin stain revealed an aggravated lens fiber defect in 8-week-old heterozygous and homozygous rncat mice in the absence of the *Hsf4* gene. Bar, 50 um.
